# Stress-Induced Accumulation of HnRNP K into Stress Granules

**DOI:** 10.26502/jcsct.5079129

**Published:** 2021-10-15

**Authors:** Jayoung Kim, Austin Yeon, Woong-Ki Kim, Khae-Hawn Kim, Takbum Ohn

**Affiliations:** 1 Departments of Surgery and Biomedical Sciences, Cedars-Sinai Medical Center, Los Angeles, CA, USA; 2 Samuel Oschin Comprehensive Cancer Institute, Cedars-Sinai Medical Center, Los Angeles, CA, USA; 3 University of California Los Angeles, CA, USA; 4 Department of Urology, Ga Cheon University College of Medicine, Incheon, South Korea; 5 Department of Microbiology and Molecular Cell Biology, Eastern Virginia Medical School, Norfolk, VA, USA; 6 Department of Urology, Chungnam National University Sejong Hospital, Sejong, Republic of Korea; 7 Department of Cellular &Molecular Medicine, College of Medicine, Chosun University, Gwangju, Republic of Korea

**Keywords:** hnRNPK, Stress granules, Apoptosis, Erk/MAPK

## Abstract

Stress granules (SGs) are cytoplasmic aggregates to reprogram gene expression in response to cellular stimulus. Here, we show that while SGs are being assembled in response to clotrimazole, an antifungal medication heterogeneous nuclear ribonucleoprotein (hnRNP) K, an RNA-binding protein that mediates translational silencing of mRNAs, is rapidly accumulated in SGs in U-2OS osteosarcoma cells. Forced expression of hnRNP K induces resistance to clotrimazole-induced apoptosis. Erk/MAPK is transiently activated in response to clotrimazole, and pharmacological suppression of the Erk/MAPK pathway sensitizes the cells to apoptosis. Inhibition of the Erk/MAPK pathway promotes the assembly of SGs. These results suggest that dynamic cytoplasmic formation of SGs and hnRNP K relocation to SGs may be defensive mechanisms against clotrimazole–induced apoptosis in U-2OS osteosarcoma cells.

## 1. Introduction

Heterogeneous nuclear ribonucleoproteins (hnRNPs) play important roles both in DNA-related functions, such as transcription, recombination, and regulation of telomere length, and in RNA-related functions, such as regulation of splicing, pre-mRNA 3’-end processing, export of mRNA from the nucleus, translation, transport of mature mRNA, and mRNA turnover [1, 2]. There are approximately 20 major hnRNPs (hnRNP A1 to hnRNP U), and the location and function of each member in various cell types are distinctive [3–5]. Some hnRNPs, such as hnRNP A, hnRNP D, hnRNP E, hnRNP I, hnRNP K, and hnRNP L, shuttle between the nucleus and cytoplasm, while others mostly exist in the nucleus [6–8].

HnRNP K is an abundant and ubiquitous protein that interacts with a diverse group of molecules [2, 9]. The function of hnRNP K is modified in response to cytokines, growth factors, oxidative stress, etc. [10]. HnRNP K is also involved in multiple processes that control gene expression [11, 12]. Previous reports demonstrated altered expression and localization of hnRNP K in human tumors, including myelogenous leukemia [13] and colorectal cancer [14, 15], suggesting the importance of mRNA metabolism regulated at the (post) translational level in cancer cells. Overexpression of hnRNP K is associated with increased transcriptional activity of oncogene c-myc and poorer survival outcomes [12], suggesting that it may have an important role in tumorigenesis. HnRNP K not only interacts with RNA, DNA, and other proteins; it also binds to factors involved in signal transduction, including mitogen activated protein kinases (MAPKs) [16]. Erk/MAPK-dependent hnRNP K phosphorylation is needed for translocation from the nucleus to cytoplasm, leading to the translational inhibition of 15-lipoxygenase. Although localization of hnRNP K is dependent on cell types, it should be noted that it may have unique motifs for nuclear/cytoplasmic shuttling. This shuttling activity of hnRNP K may be essential for biological responses that control cellular differentiation, proliferation, and survival [9, 17–20]. Electron microscopic examination revealed that hnRNP K exists in the nucleus, cytoplasm, mitochondria, and within the vicinity of the plasma membrane [21]. Interestingly, nucleus-residing hnRNP K in colon cancer cells was found to be associated with increased survival rates [22].

Post-transcriptional regulation of gene expression upon various stimuli, such as heat shock, oxidative stress, viral infection, is vital for cell survival [23]. Stress granules (SGs) are cytoplasmic sites in which translationally stalled mRNAs and numerous RNA binding proteins are nucleated upon stresses [24], and this event allows cell to reprogram gene expression [25]. SGs are signaling platforms that contribute to the coordination of cellular processes. The core constituents of SGs are small ribosomal subunits, translation initiation factors (e.g., eIF4E, eIF3, eIF4G, and PABP), and various RNA binding proteins that regulate translation or mRNA decay [26]. It has been suggested that SGs are the sites where mRNA triage takes place to direct RNAs to be degraded or re-translated. A recent study showed that SGs also contain micro-RNA machinery, suggesting a possible link between these two pathways [27]. The SG components that contribute to the cellular responses to stress stimuli remain elusive despite recent advances in purification and molecular profiling technologies [28, 29]. In this study, we tested the hypothesis that hnRNP K is recruited to SGs in response to apoptotic stimuli, which is an essential survival mechanism. We induced apoptosis of the U-2OS osteosarcoma cells by acute treatment with clotrimazole, a broad-spectrum antimycotic drug mainly used for the treatment of fungal infections. We further tried to understand the key signaling pathways required for defense mechanism against clotrimazole-induced apoptosis.

## 2. Materials and Methods

### 2.1 Antibodies and reagents

The antibodies used in this study include the followings: anti-hnRNP K (sc-28380) and anti-EIF3α (sc-376651) (Santa Cruz Biotechnology, Santa Cruz, CA), anti-phospho-Erk/MAPK (9101), anti-Erk/MAPK (9102), anti-HA-Tag (3724), anti-GAPDH (5174), anti- β-Tubulin (2146), and anti-Lamin A/C (4777) (Cell Signaling Technology, Beverly, MA). A specific MEK1 inhibitor, PD98059 (513000), and p38MAPK inhibitor, SB203580 (559389), were purchased from Sigma-Aldrich (St. Louis, MO). All other chemicals, including clotrimazole, were obtained from Sigma-Aldrich.

### 2.2 Cell culture and transfections

The U-2OS osteosarcoma cell line was procured through American Type Culture Collection and was maintained in Dulbecco’s modified Eagle’s medium (DMEM) (high glucose), 10% fetal bovine serum, 100μg streptomycin, and 100 units/ml penicillin (Invitrogen, Carlsbad, CA) at a humidified atmosphere of 5% CO_2_ at 37°C. U-2OS cells in 150 mm dishes at ~80% confluence were applied to electroporation with an empty vector or a hnRNPK expressing plasmid using nucleofector (Amaxa Inc., Gaithersburg, MD) followed by instructions supplied by the company. For siRNA transfection, cells were cultured in 6well plates at a density of 1×10^5^cells/mL. After 24 h, cells at ~80% confluence were transiently transfected with 5-nM small interfering RNAs (siRNAs) of hnRNPK (SigmaAldrich) or negative control siRNA (siCTL) (Ambion, Austin, TX, USA), by using Lipofectamine RNAiMAX (Thermo Fisher Scientific Inc., Carlsbad, CA, USA), according to the manufacturer’s instructions. As transfection controls, empty vector or NON- TARGET control siRNAs were used. Mock cells were treated with RNAiMAX and cultured in Opti-MEM for 6 hrs, but without siRNA.

### 2.3 Preparation of whole cell lysates and immunoblot analysis

Treated cells were washed twice in ice-cold phosphate-buffered saline (PBS) and lysed in a minimum volume of 1X cell lysis buffer [1% Nonidet P-40; 50 mM Tris pH 7.4; 10 mM NaCl; 1 mM NaF; 5 mM MgCl_2_; 0.1 mM EDTA; 1 mM PMSF; and COMPLETE™ protease inhibitor cocktail tablet (Roche Diagnostocs Corp.)]. Protein content was determined using the Micro BCA Protein Assay Kit (Thermo Scientific, Rockford, IL). Cell extracts (10 μg/lane) were resolved by 4–12% gradient SDS-polyacrylamide gel electrophoresis (Bio-Rad, Hercules, CA) and electro-transferred onto nitrocellulose membranes. Following the transfer, membranes were stained with Ponceau S to confirm equal protein loading. Membranes were blocked with PBS/0.1% Tween-20 (PBST) and 10% skim milk and incubated with antibodies in PBST overnight at 4°C. Following incubation with species-specific horseradish peroxidase (HRP)-conjugated secondary antibodies, signals were detected using the SuperSignal Chemiluminescent Reagent (Pierce Chemical Co., Rockford, IL) with exposure of blots onto X-ray films.

### 2.4 Cell proliferation assay and apoptosis analysis

The proliferation rate was determined by counting cell numbers under the indicated conditions. Fluorescence-activated cell sorting (FACS) analysis was performed to verify the apoptotic cell population by measuring the subG_o_ population. After harvesting at the indicated conditions, cells were stained with propidium iodide, and visualized by flow cytometry. Cell proliferation assays using 3-(4, 5dimethylthiazolyl-2)-2, 5-diphenyltetrazolium bromide (MTT), and cell viability assays using crystal violet staining were performed to determine cell numbers. All experiments were performed in 6 biological replicates and mean values were calculated. TUNEL assay was performed to compare apoptotic levels in response to clotrimazole with or without PD98059, a MEK1 inhibitor, or SB203580, a p38MAPK pathway inhibitor. Cells in the cover slip were incubated in PD98059-containing medium for 1 h, followed by treatment with 20 μM clotrimazole for an additional 30 min.

### 2.5 Immunofluorescence microscopy

For imaging experiments, 1 × 10^3^ cells were plated on glass cover slides (VWR, West Chester, PA) 24 h before drug treatment. Cells with 80% confluency were used for the following experiments. Pre-incubation of cells with 50 μM PD98059 for 1 h was followed by treatment with 20 μM of clotrimazole in serum-free medium. Immunostaining was done using the following primary antibodies after clotrimazole treatment: anti-EIF3α pAb (SGs marker), or anti-hnRNP K mAb at dilutions of 1:100, 1: 50, and 1:100, respectively. Cells were fixed with 4% PFA formaldehyde for 15 min followed by ice-cold methanol for 5 min. Cells were then washed once with ice-cold PBS, and non-specific binding sites were blocked in PBS/0.1% BSA for 1 h at room temperature prior to incubation with primary antibodies. The immune reaction for each primary antibody was detected by Cy5 (blue; for EIF3α), or FITC-(green; for hnRNPK) conjugated secondary antibodies (1:250) for 30 min at room temperature. Slides were mounted in Vectashield medium-containing DAPI (Vector Laboratories, Inc., Burlingame, CA) and analyzed using AxioVision under a microscope (Carl Zeiss Inc.).

### 2.6 Statistical analysis

All experiments were repeated in 6 biological duplicates for statistical analysis. The data were expressed as mean ± standard deviation (SD) for continuous variables while frequencies (%) for categorical variables. Students’ t test and one-way ANOVA post-hoc Tukey’s test were used to compare the data from different groups. *P* < 0.05 was considered statistically significant.

## 3. Results

### 3.1 Clotrimazole treatment induced formation of SGs and apoptosis in U-2OS sarcoma cells

**Figure 1:**
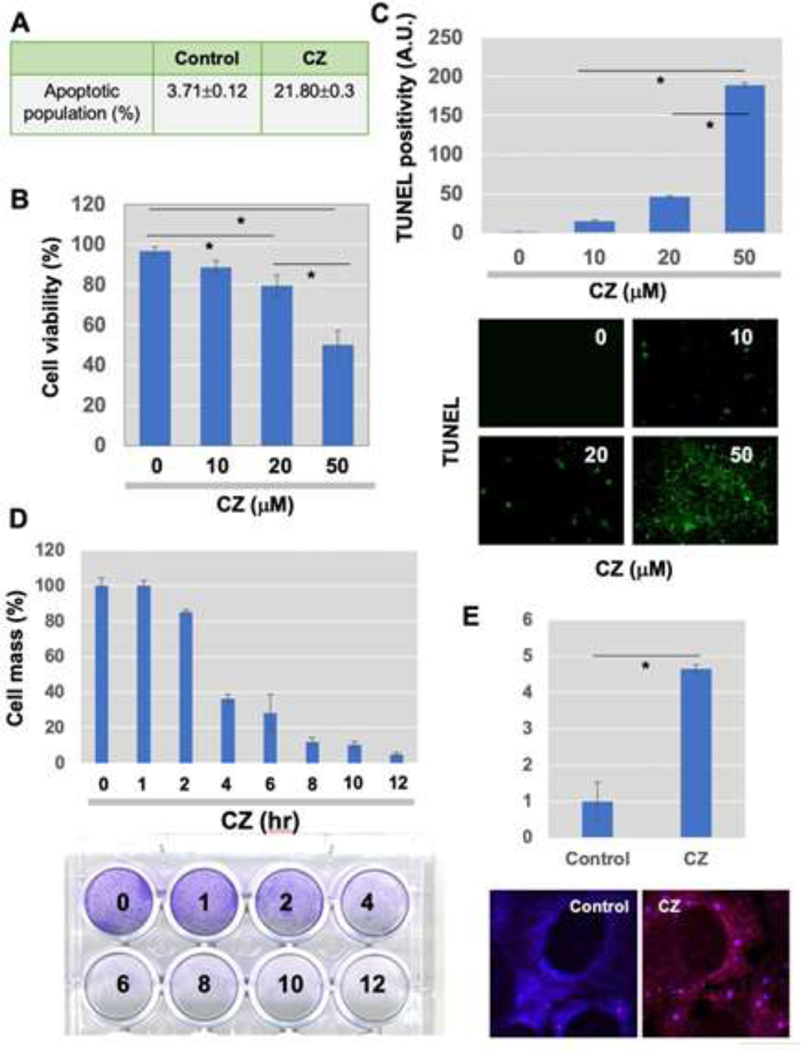
Clotrimazole treatment-induced assembly of stress granules (SGs), followed by apoptosis in U-2OS osteosarcoma cells. (A) U-2OS cells were incubated in serum-free medium containing 20 μM clotrimazole for 6 h. To measure apoptotic cell population, FACS analysis was performed after fixation and staining of cells; (B and C). (B) MTT-based proliferation assay 24 h after treatment of cells with 20 μM clotrimazole and (C) TUNEL assay were done to determine proliferation and apoptosis in response to clotrimazole (Green in Figure 1C, apoptotic cells). Bar graph representing the percentage of apoptotic cells. Error bars indicate standard errors. (n=6). *P<0.01. (D) U-2OS cells pretreated with 20 μM clotrimazole for indicated time points (0, 1, 2, 4, 6, 8, 10, and 12 h) and crystal violet staining was performed. (E) Immunofluorescence (IF) staining analysis using marker proteins for SGs was performed 30 min after 20 μM clotrimazole treatment in serum-free medium, which was further processed for IF microscopy. The fold change of the % of accumulation into foci was shown. Scale bar, 10 μm.

Tight control of translation is fundamental in cellular homeostasis for eukaryotic cells, and deregulation of proteins contributes to numerous human diseases. SGs and processing bodies (PBs) are the main intracellular compartments for regulating and controlling mRNA degradation, stability, and translation, which are involved in many biological responses including cell proliferation, differentiation, apoptosis, and development [26, 30, 31]. We sought to examine in this study whether apoptosis induced by clotrimazole is linked to the functional formation of SGs, and whether hnRNPK, a potential translational regulator, is modulated during granule assembly. Clotrimazole, an antifungal drug that dissociates Hex II from the mitochondria [32], significantly induces apoptosis in U-2OS osteosarcoma cells. FACS analysis revealed that about 21.8% of cells went into the apoptotic phase 6 h after treatment with 20 μM clotrimazole (Figure 1A). MTT assays showed a significant, dose-dependent reduction of cellular proliferation with clotrimazole (Figure 1B). TUNEL assays demonstrated increased numbers of apoptotic (green) cells detected in a dose-dependent manner (Figure 1C). When cell proliferation was measured via crystal violet staining, proliferation dramatically decreased in a time- dependent fashion, particularly with treatment with 20 μM clotrimazole for 4 h (Figure 1D).

### 3.2 HnRNP K is necessary as a defensive mechanism against clotrimazole-induced apoptosis

U-2OS cells formed RNA granules, such as SGs, within 30 min of being treated with clotrimazole. This was observed with immunofluorescence (IF) staining of EIF3α, which indicates SGs. Representative stained images of normal and clotrimazole-stimulated conditions are shown in Figure 1E. This data demonstrated that SGs are rapidly translocated specifically to the cytoplasmic foci.

**Figure 2:**
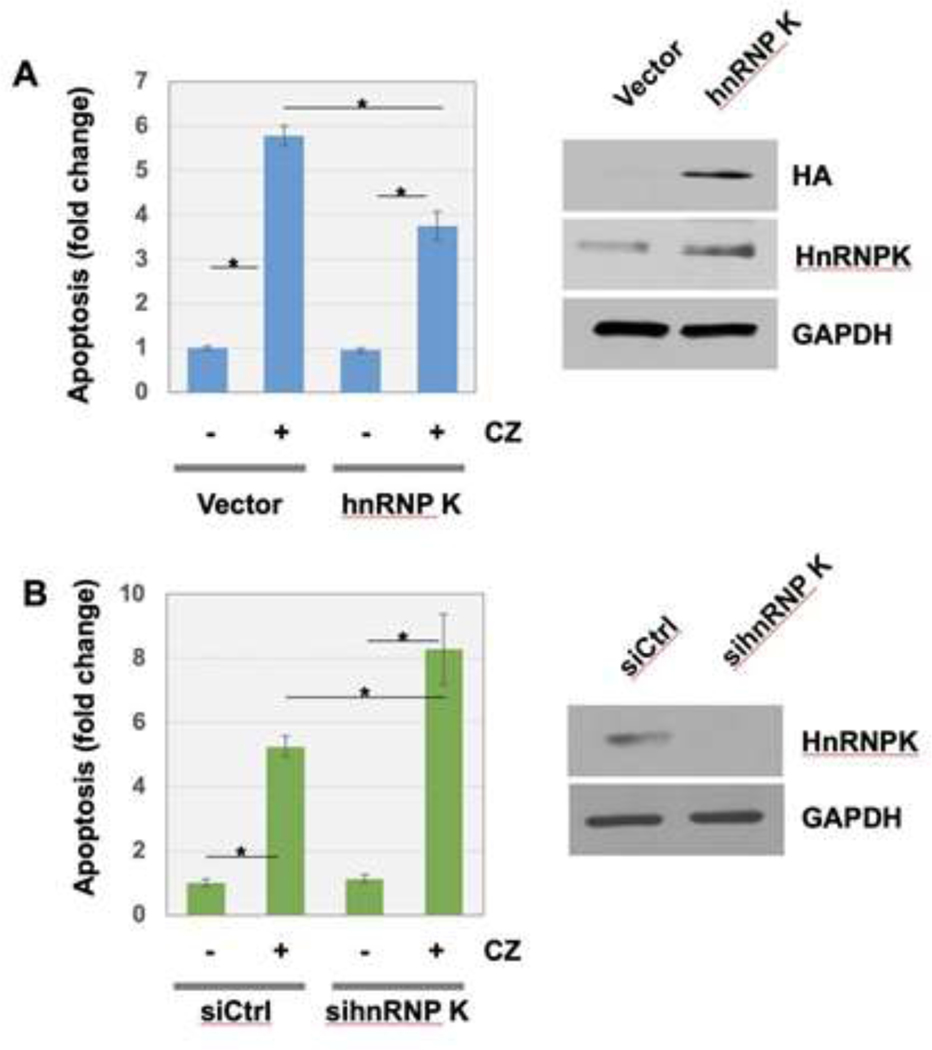
HnRNP K suppressed clotrimazole-induced apoptosis of U-2OS cells. (A) Ectopic expression of hnRNP K overexpressing construct or vector only in U-2OS cells was followed by clotrimazole treatment. The expression of HA-HnRNP K constructs were assessed by Western blot analysis using anti-HA or anti-HnRNP K antibodies. (B) U-2OS cells transiently transfected with control siRNA or si hnRNP K were treated with clotrimazole. For A and B, FACS analysis using 6 biological replicates was performed to determine apoptosis level at the indicated conditions. Error bars indicate standard derivation. *P<0.01.

In addition, we found that cells harboring ectopic hnRNP K were more resistant to the clotrimazole-induced apoptosis, compared to cells transfected with vector plasmid (Figure 2A). In control condition, clotrimazole treatment increased apoptosis approximately 6-fold. Overexpression of hnRNP K made U-2OS cells approximately 35% more resistant to the apoptosis induced by clotrimazole. The efficient overexpression of hnRNP K were confirmed, which was shown by Western blot analysis using anti-HA tag and anti-hnRNP K (Figure 2A, right panels). When hnRNP K expression was silenced with siRNA transfection, cells were approximately 140% more sensitized to clotrimazole treatment compared to control (Figure 2B). The knock down of hnRNP K by siRNA transfection were confirmed, which was shown by Western blot analysis using anti-hnRNP K (Figure 2B, right panels).

### 3.3 HnRNP K is recruited to cytoplasmic SGs in response to clotrimazole

**Figure 3:**
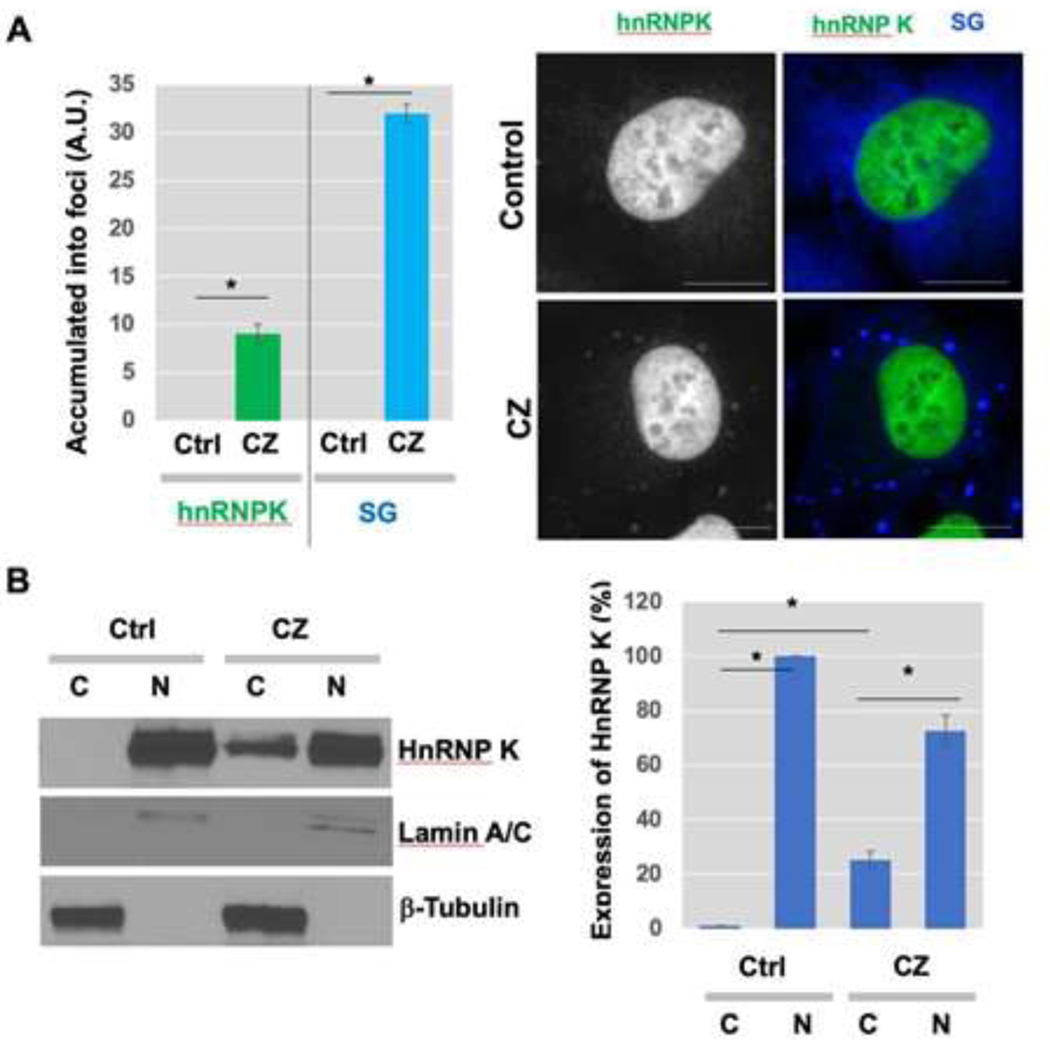
HnRNP K translocated into SGs with clotrimazole treatment. (A) Cells were incubated in serum-free medium in presence of 20 μM clotrimazole for 30 min. After cell fixation with 4% PFA solution and methanol, cells were stained using various antibodies, as described in Materials and Methods (Gray/Green, hnRNP K; Blue, EIF3α). Scale bar, 10 μm. (B) Cytosol accumulation of hnRNP K. U-2OS cells were treated with clotrimazole for 2 h. Western blot analysis using anti-Lamin A/C and anti-β-Tubulin was performed and the successful subcellular fractionation for nuclear and cytoplasmic fractions was confirmed, respectively. Same concentration of proteins were used for the following Western blot analysis to measure the protein expression levels of hnRNP K in cytoplasmic vs nuclear fractions. ImageJ software was used to quantify the band intensities to determine the ratio of nuclear vs cytoplasmic hnRNP K (right).

Examination of SGs in apoptotic human sarcoma cells showed that hnRNP K, which is predominantly localized to the nucleus normally, exhibited translocation upon clotrimazole treatment (Figure 3A). SGs are rapidly assembled and accumulated as cytoplasmic foci in response to clotrimazole (blue) (Figure 3A, right). These findings may suggest that translocation of hnRNP K to SGs in the cytosol could be related to the function of hnRNP K in the regulation of general translation under stress conditions. To further test the translocation of hnRNP K from the nucleus to cytosol in response to clotrimazole, cells were treated with clotrimazole and the expression of nuclear and cytoplasmic hnRNP K was examined. Subcellular fractionation and Western blot analysis showed that some part of endogenous hnRNP K (approximately 25%) moved from the nucleus to cytoplasm (Figure 3B, left). Quantitative data showing the expression % of hnRNP K in nuclear vs cytoplasmic fractions were shown in the graph (Figure 3B, right).

### 3.4 Suppression of Erk/MAPK sensitizes clotrimazole-induced apoptosis

**Figure 4:**
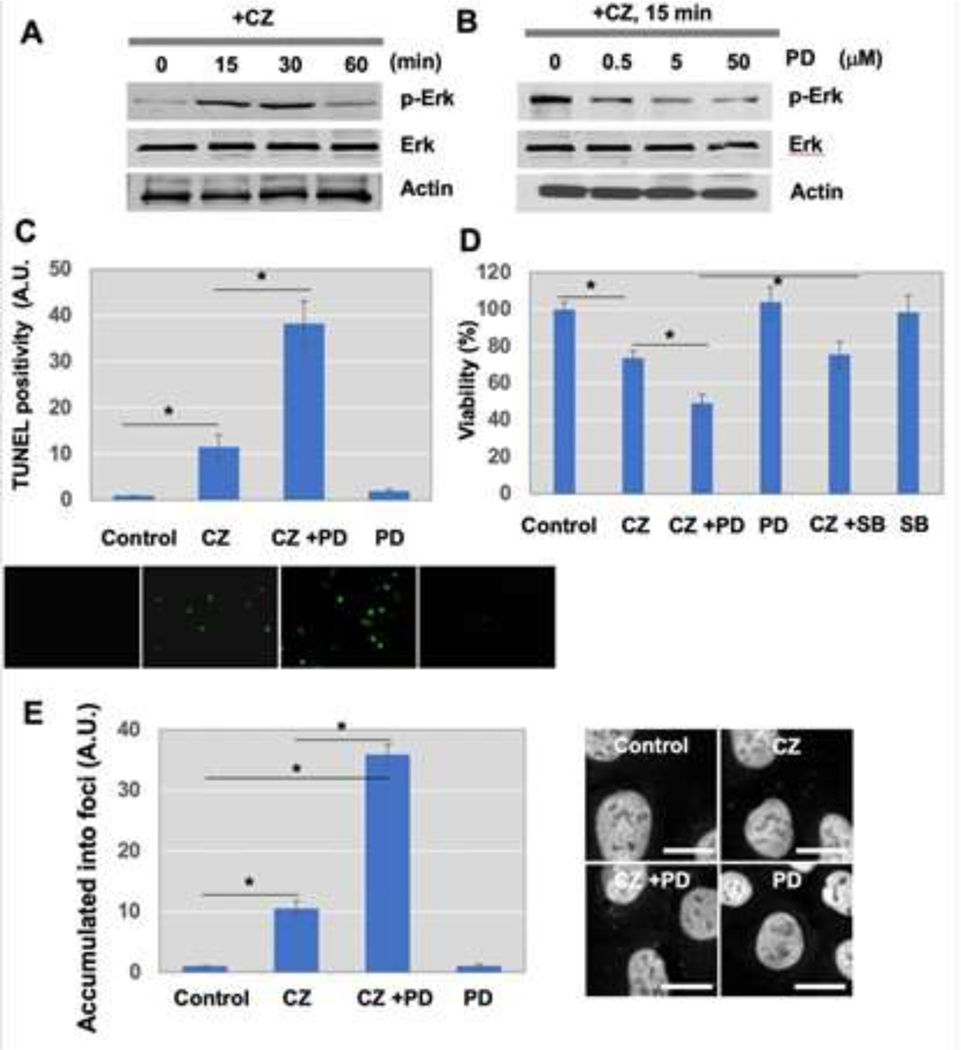
Erk/MAPK activation by clotrimazole treatment suppressed apoptosis, but not trafficking of hnRNP K. (A) Cells were treated with 20 μM clotrimazole for the indicated times. Western blot analysis was performed to assess phosphorylation status of Erk/MAPK and p38MAPK in response to clotrimazole. The protein expression level of total Erk/MAPK and p38MAPK was also determined. (B) Inhibition of MEK1, an upstream molecule of Erk/MAPK, with PD98059 suppressed Erk/MAPK phosphorylation after treatment with clotrimazole. Cells were pretreated with 10 μM PD098059 for 30 min and stimulated with clotrimazole for 15 min. (C) Blockage of Erk/MAPK by PD98059 increased cell apoptosis by clotrimazole. TUNEL assay was used for determination of apoptosis. Green spots indicate apoptotic cells. Representative images were shown. (D) To observe the increased apoptosis by inhibition of Erk/MAPK or p38MAPK, cells were seeded with the same density 1 day before clotrimazole treatment. Cells were pretreated with 10 μM PD098059 or 10 μM SB203580 for 30 min and stimulated with clotrimazole for 2 h. Cells were stained by crystal violet solution after fixation and stained cells in purple were counted as viable. (E) Cells were pretreated with 10 μM PD98059 or 10 μM SB203580 for 30 min, followed by incubation with 20 μM clotrimazole for 15 min. Stained images of hnRNP K were analyzed under microscopy as described in Materials and Methods. Scale bar, 10 μm.

To investigate signal transduction pathways involved in the assembly of SGs after treatment with clotrimazole, several signal pathways were examined. Western blot analysis using anti-phospho-Erk/MAPK antibodies demonstrated that Erk/MAPK is activated transiently 15 min after clotrimazole treatment (Figure 4A), while p38MAPK was not activated (Figure 4A). Protein levels of Erk/MAPK were not affected by clotrimazole (Figure 4A). Activation of the Erk/MAPK pathway has been linked to enhanced proliferation and anti- apoptosis of tumor cells [33].

In experiments aimed at manipulating this pathway, we used a selective inhibitor of MEK1, PD098059, and assessed the involvement of the Erk/MAPK pathway in increased apoptosis after clotrimazole treatment. Phosphorylation levels of Erk/MAPK were diminished in the presence of PD098059 (Figure 4B). Efficacy of the inhibitor was monitored by its ability to block phosphorylation of Erk/MAPK, while levels of total Erk/MAPK were not changed (Figure 4B). Both TUNEL assays (Figure 4C) and cell viability analysis (Figure 4D) showed that suppression of the Erk/MAPK pathway enhanced apoptosis induced by clotrimazole. These results suggest that activated Erk/MAPK plays as a survival mechanism for cells against clotrimazole-induced apoptosis.

In contrast to apoptosis induction, further examination using IF staining analysis revealed that hnRNP K localization to SGs corresponded to Erk/MAPK activation (Figure 4E). The hnRNP K accumulation to foci was stimulated in response to clotrimazole treatment (CZ), which was significantly enhanced when Erk/MAPK was inhibited (CZ+PD) (Figure 4E). There was no significant change in PD98059 (PD) only, compared to control (Con). Inhibition of the p38MAPK pathway by a specific inhibitor, SB203580, had no effect on hnRNP K accumulation to foci (Figure 4E). Taken together, these experiments suggest the role of the Erk/MAPK pathway as a main mediator of clotrimazole-stimulated cell apoptosis and the formation/ accumulation of SGs, but not for the formation/ accumulation of PBs in U-2OS cells.

## 4. Discussion

Herein, we provide evidence that hnRNP K is translocated to cytoplasmic SGs in response to apoptotic stress induced by clotrimazole in U-2OS sarcoma cells, and that the Erk/MAPK signal pathway is activated but not required for this phenomenon. Our study is the first to address the potential role of SGs in trafficking hnRNP K in human cancer cells. SGs harbor various RNA-binding proteins and mRNAs, which play vital roles in alternative splicing. SGs are a platform of mRNA trafficking to processing bodies (PBs), where mRNA decay occurs. Our results showed that hnRNP K is present in SGs, suggesting that (1) hnRNP K binds to mRNAs to be degraded or protected from mRNA degradation upon stress stimuli, and (2) mRNA turnover can be regulated by foci formation. Microscopic examination revealed that hnRNP K accumulates dramatically and rapidly, within 30 min, to RNA granules. Being a transient phenomenon, this was consistent with previous observations that once stress is relieved, SGs disassemble [24, 34, 35]. This also supports the idea that formation of RNA granules is important in tight regulation of gene expression in response to stress. However, the complete mechanism of how these cytoplasmic foci is assembled is unknown. Our microscopic images also showed that many SGs overlap or at least assemble closely. Since the functions of SGs are known to be distinctive, we speculated that hnRNP K in SGs would move to PBs under specific conditions via tight communication between SGs and PBs.

Cells respond to stress stimuli by activating defensive survival mechanisms to prevent damage to some extent, and, when necessary, activate apoptosis. Among the MAPK pathways, the JNK/SAPK and p38MAPK pathways are considered to play major roles during apoptosis in response to stress stimuli. The activation of signaling pathways regulate the subcellular distribution of RNA-binding proteins and mRNA decay. The hnRNP A1, a nucleocytoplasmic shuttling protein, is translocated into SGs depending on p38MAPK and Mnk1/2-involved phosphorylation [36]. Phosphorylation of hnRNP K at serine 284 and 353 by serum-induced Erk/MAPK activation results in enhanced cytoplasmic translocation of hnRNP K and suppressed mRNA translation [16]. Our data showed that clotrimazole treatment activates Erk/MAPK, but not p38MAPK. Inhibition of Erk/MAPK or p38MAPK affect the accumulation of hnRNP K into cytoplasmic foci upon treatment with clotrimazole. However, we cannot rule out the possibility that clotrimazole may stimulate other signaling pathways resulting in the accumulation of hnRNP K into RNA granules.

Furthermore, our study found that cytoplasmic accumulation of hnRNP K is crucial for its role in metastasis by functional interference screening. Our data showed that forced expression of hnRNP K suppressed apoptosis that is induced in response to clotrimazole treatment, suggesting that hnRNP K plays an important role in stress-induced survival pathways. This observation is consistent with the previous report suggesting hnRNP K as a potential target to halt cancer progression. Although the specific role of hnRNP K sequestering to these foci is not clearly understood, it is possible that recruitment of hnRNP K to SGs may be of wider significance, considering it modulates gene expression and translation metabolism.

## List of abbreviations:

DMEM: Dulbecco’s modified Eagle’s medium
FACS: Fluorescence-activated cell sorting
hnRNPs: Heterogeneous nuclear ribonucleoproteins
IF: Immunofluorescence
MAPKs: Mitogen activated protein kinases
siCTL: siRNA Control
SGs: Stress granules
SD: Standard deviation
PBs: Processing bodies
MTT: 3-(4, 5-dimethylthiazolyl-2)-2, 5-diphenyltetrazolium bromide
